# Electrochemical water oxidation by simple manganese salts

**DOI:** 10.1038/s41598-019-44001-z

**Published:** 2019-05-23

**Authors:** Sima Heidari, Jitendra Pal Singh, Hadi Feizi, Robabeh Bagheri, Keun Hwa Chae, Zhenlun Song, Maasoumeh Khatamian, Mohammad Mahdi Najafpour

**Affiliations:** 1 Department of Inorganic Chemistry, Faculty of Chemistry, University of Tabriz, Tabriz, Iran; 2 Advanced Analysis Center, Korea Institute of Science and Technology, Seoul, 02792 Republic of Korea; 3 Department of Chemistry, Institute for Advanced Studies in Basic Sciences (IASBS), Zanjan, Iran; 4 Surface Protection Research Group, Surface Department, Ningbo Institute of Materials Technology and Engineering, Chinese Academy of Sciences, 519 Zhuangshi Road, Ningbo, 315201 China; 5 Center of Climate Change and Global Warming, Institute for Advanced Studies in Basic Sciences (IASBS), Zanjan, Iran; 6 Research Center for Basic Sciences & Modern Technologies (RBST), Institute for Advanced Studies in Basic Sciences (IASBS), Zanjan, 45137-66731 Iran

**Keywords:** Electrochemistry, Inorganic chemistry

## Abstract

Recently, it has been great efforts to synthesize an efficient water-oxidizing catalyst. However, to find the true catalyst in the harsh conditions of the water-oxidation reaction is an open area in science. Herein, we showed that corrosion of some simple manganese salts, MnCO_3_, MnWO_4_, Mn_3_(PO_4_)_2_ · 3H_2_O, and Mn(VO_3_)_2_ · xH_2_O, under the water-electrolysis conditions at pH = 6.3, gives an amorphous manganese oxide. This conversion was studied with X-ray absorption spectroscopy (XAS), as well as, scanning electron microscopy (SEM), Energy-dispersive X-ray spectroscopy (EDXS), transmission electron microscopy (TEM), X-ray photoelectron spectroscopy (XPS), X-ray diffraction (XRD), spectroelectrochemistry and electrochemistry methods. When using as a water-oxidizing catalyst, such results are important to display that long-term water oxidation can change the nature of the manganese salts.

## Introduction

Electrochemical water splitting to hydrogen and oxygen is a promising way to generate hydrogen fuel^1^. According to thermodynamic and kinetic barriers, the oxygen-evolution reaction (OER) is the most challenging half-reaction in the water splitting^2^. Hence, developing efficient water-oxidizing catalysts with lowering the overpotential of the oxidation half-reaction and thus expedite the reaction is an active area in science^2–7^. Due to the great amount of the current needs to energy sources, design and synthesis of inexpensive water-oxidizing catalysts based on earth-abundant materials are essential. Therefore, first-row transition metals and their derivatives are among the most promising water-oxidizing catalysts for future^3^.

Among different transition-metal ions, manganese compounds have attracted tremendous attention. Because a Mn_4_CaO_5_ cluster in photosystem II (PSII) is the only natural system that demonstrates water oxidation^8^, manganese-based compounds from solid-state materials to molecular complexes have been potentially interesting materials for water oxidation in the past few decades^2,9–13^. Manganese is also an earth-abundant, environmentally friendly and low-cost element. Interesting properties of manganese oxides have made them attractive materials in various catalytic applications and energy storages, as well^14–22^.

There have been considerable efforts to find efficient manganese complexes as homogenous or heterogeneous water-oxidizing catalysts^12,13^, it has been established that in most cases these compounds are unstable upon operation under harsh conditions of chemical or electrochemical water oxidation and are converted to manganese oxides^23–28^. Not only the conversion of manganese complexes to oxide phases but also surface amorphization of crystalline calcium manganese oxides or manganese oxides in the chemical or electrochemical water oxidation reaction has been reported^29–31^. These converted materials showed exceed catalytic activity^29–31^. This process is also the reason for the instability of mixed oxide systems such as Ba_0.5_Sr_0.5_Co_0.8_Fe_0.2_O_3−δ_ and La_0.65_Sr_0.35_MnO_3_ during the OER^32,33^. Comparably, an amorphous phase has been reported as the active phase in the water-oxidizing activity of a series of cobalt-substituted crystalline zinc oxide precatalysts^34^. Additionally, it was found that in the water-oxidizing activity of cobalt selenide particles, an amorphous cobalt oxide was the true catalyst^35^. Reports from Hu’s lab displayed an *in situ* formation of a core-shell Ni_2_P/NiO_x_ assembly during the water-oxidation performance of Ni_2_P catalyst^36^. Likewise, reorganization of the material to an active amorphous phase under catalytic condition was responsible for water-oxidizing activity of CoP precatalysts^37^.

All above-mentioned cases highlight the importance of studying the long-term stability of the water-oxidizing catalysts. Especially finding the true catalysts, is a key step in the commercial application of a water-oxidizing catalyst. Finding the reason for the instability of a catalyst and identifying the true catalyst in the applied condition is also helpful to design and synthesize efficient water-oxidizing catalysts with higher activity, and better stability. Recently, we showed that a series of non-oxide manganese salts are converted to an amorphous manganese oxide phase, in the presence of Ce(IV) solution as an oxidant^38^. Herein, we studied the long-term stability of a series of manganese salts (MnCO_3_, MnWO_4_, Mn(VO_3_)_2_ · xH_2_O and Mn_3_(PO_4_)_2_ · 3H_2_O), under the electrochemical water-oxidation conditions. This question may rise why studying the stability of these materials is important under both chemical and electrochemical water oxidation condition. Under chemical water-oxidation conditions in the presence of an oxidant, the high oxidizing ability of the oxidant, as well as, pH of the solution might be effective in the instability of a catalyst (the pH of a Ce(IV) solution is around 1). However, in this work, we have applied an electrochemical condition in a neutral electrolyte to eliminate the pH effect. On the other hand, despite chemical methods, the applied potential is tunable in the electrochemical method; therefore, one can investigate the effect of applied overpotential on the stability of the utilized catalyst. We could detect the transformation of the mentioned manganese salts to an amorphous manganese oxide phase using various techniques such as XRD, SEM, EDXS, HRTEM, XANES and EXAFS, XPS, spectroelectrochemistry, and electrochemistry. From now on, the freshly prepared salts and the converted materials are denoted according to Table 1.

**Table 1:** The investigated samples in this study^a^.

Sample	MnCO_3_	MnWO_4_	Mn_3_(PO_4_)_2_ · 3H_2_O	Mn(VO_3_)_2_ · xH_2_O
Before conversion	MCB	MWB	MPB	MVB
After conversion	MCA	MWA	MPA	MVA

^a^The “B” suffix means “before conversion”, and the “A” suffix abbreviates “after conversion”.

## Results

Manganese salts were synthesized using a simple precipitation method. Then, the synthesized salts were used under electrochemical water-oxidation conditions at a pH around 6.3. Therefore, manganese salts were introduced to the anodic half-cell of a convective-suspension-collision system^39^.

In this system, a half-cell consisted of a platinum plate as an electrode in a 1 M KCl solution that was used as a reference half-cell. On the other hand, a platinum plate, which was immersed in a suspension of mother salts in a 0.1 M LiClO_4_ solution acted as a working half-cell. Finally, an inverted glass U-tube filled with a mixture of 5% agar in 1 M KCl had connected two half-cells as a salt bridge. A 2.0 V potential was applied between two electrodes. The suspension in the working half-cell was stirred by using a magnetic stirrer, and the synthesized salts collided with the anode continuously. Since in this method no binder or polymer (such as Nafion) has been utilized to attach the material on the conductive electrode, we could provide a large amount of the post operando material without any contamination of the binder polymer. It should be noted that as the post operando material might be an amorphous phase, any amorphous species could influence the characterization accuracy. Therefore, to have a precise detection on the post catalytic species, avoiding such amorphous contamination was very important.

After the conversion of the mother salt under the convective-suspension-collision condition for 24 h, a brown solid was precipitated on the surface of the anode. The brown compound was scratched and was analyzed with different methods. In the case of MPB, the conversion happened slowly. Thus, we collected the brown precipitate after 5 days.

The characterization of the post operando materials showed that the investigated salts were converted to an amorphous manganese oxide phase with different morphology and an unlike chemical structure to mother compounds.

**Figure 1:**
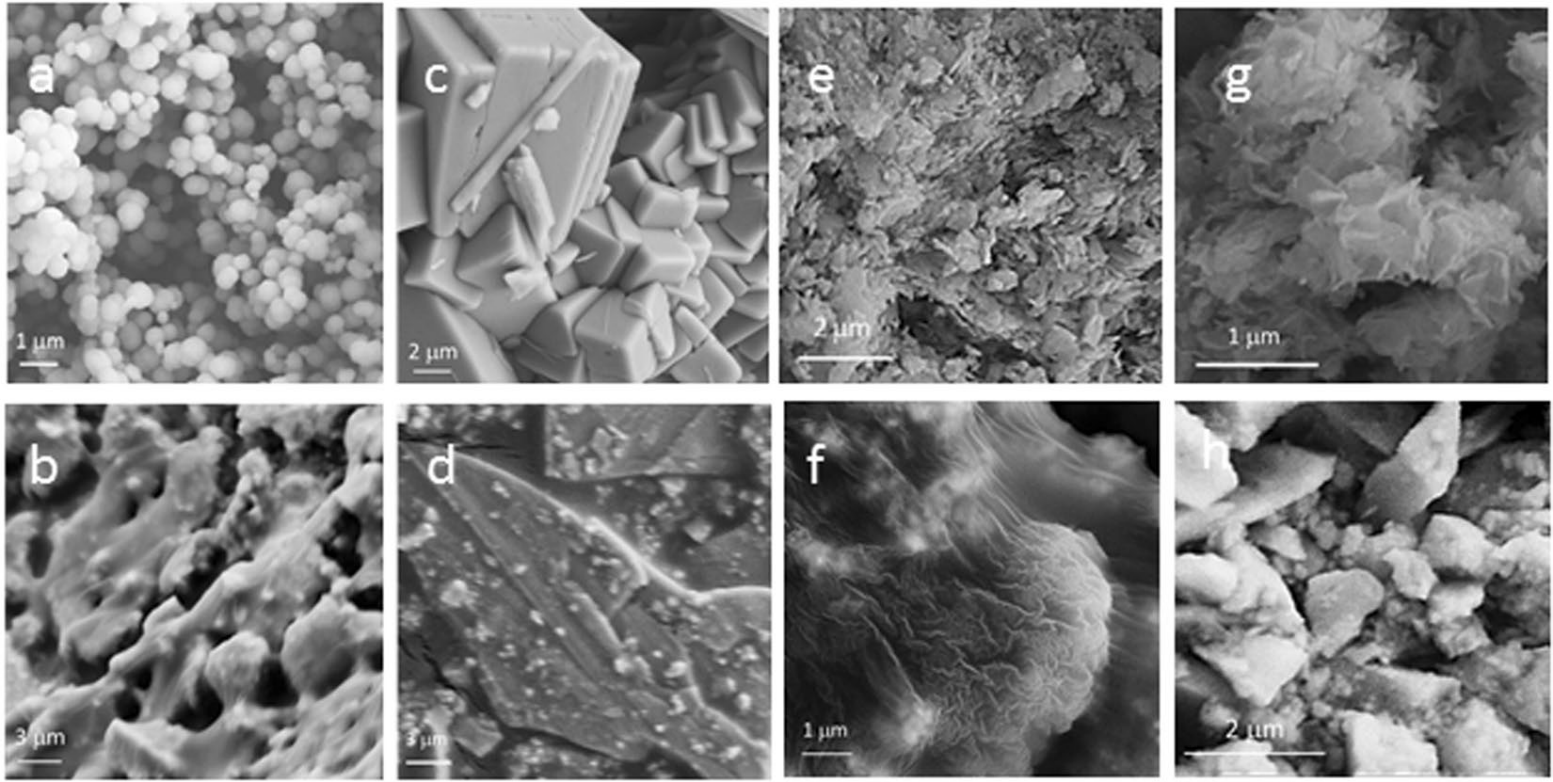
SEM images of MCB (**a**) MCA (**b**) MVB (**c**) MVA (**d**) MPB (**e**) MPA (**f**) MWB (**g**) and MWA (**h**). SEM images show that while all mother salts have different morphologies, the converted materials display no distinguishable morphology, which suggested the low crystalline structure of them.

The morphologies of the different fresh manganese salts and the samples after the conversion were studied using SEM (Fig. 1). While MPB, MWB, MVB, and MCB samples showed various morphologies, all the converted materials exhibited no distinguishable morphology in SEM imaging, which suggested the low crystalline structure of them.

Since the oxygen evolution reaction occurs on the surface of a catalyst rather than the bulk of the sample, the samples were studied with EDXS analysis, as well. EDXS results of the original salts and the converted materials have been presented in Table 2.

**Table 2:** EDXS results for the investigated compounds in this research^a^.

Element	Mn	P	O
Sample			
MPB	44.13	18.08	37.79
MPA	26.4	25.11	45.02
**Element**	**Mn**	**V**	**O**
**Sample**			
MVB	15.82	33.96	41.00
MVA	14.50	0.00	33.34
**Element**	**Mn**	**W**	**O**
**Sample**			
MWB	19.25	40.84	11.92
MWA	32.53	6.05	24.60

^a^According to the inaccuracy of the EDXS results for carbon, this analysis was not performed on MCB and MCA samples.

Interestingly, vanadium content in the EDX results for MVA is zero, which confirms, at least in the interaction volume of the analysis; whole manganese vanadate was converted to manganese oxide in the electrocatalytic reaction. Pourbaix diagram displays that vanadium ions could form soluble species under this condition^40^.

**Figure 2:**
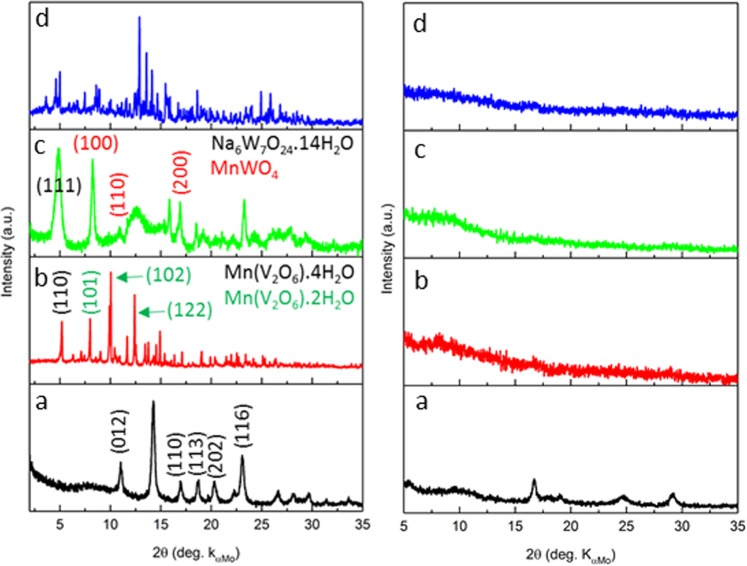
Left panel: XRD patterns of MCB (**a**) MVB (**b**) MWB (**c**) MPB (**d**). Right panel: XRD patterns of MCA (**a**) MVA (**b**) MWA (**c**) MPA (**d**). Well-defined crystalline structures are observed for all original salts. However, no recognizable diffraction peaks for the converted materials confirm the amorphous phase of these samples.

Figure 2 shows XRD patterns of all samples, before and after the electrocatalytic reaction. Full interpretations of XRD data have been presented in Figs S1–S5. The XRD pattern of MCB sample (Fig. 2a right panel), demonstrated the crystalline structure of this sample and the pattern completely match to MnCO_3_ structure. The MVB sample had two crystalline phases: MnV_2_O_6_ · 2H_2_O and MnV_2_O_6_ · 4H_2_O. No other impurities were observable in the XRD pattern of this sample. In the XRD pattern of the MWB sample in Fig. 2c (right panel), the main peaks at ~8.24°, 11.01 and 16.94° are well consistent with the manganese tungstate phase. The sharp peak at ~4.96°, which corresponds to the main peak of Na_6_W_7_O_24_ · 14H_2_O phase, shows the co-existence of this phase in the MWB sample. The XRD pattern of MPB sample contains main diffraction peaks at 2θ = 12.15, 13.53, and 15.68° which could be assigned to crystalline Mn_3_(PO_4_)_2_ · 3H_2_O phase with 3.35, 3.01 and 2.6 Å d-spacing, respectively.

As it is clear in Fig. 2, well-defined crystalline structures were observed for all original salts. However, no recognizable diffraction peaks for the converted materials corroborate amorphous phase of these samples. In the case of MCA sample, despite to the amorphous background, a few broad peaks were visible at 2θ = 5.59, 11.05, 16.73, 29.27° with 7.2, 3.6, 2.4 and 1.4 Å d-spacing which were related to a manganese oxide phase with a layered structure.

**Figure 3:**
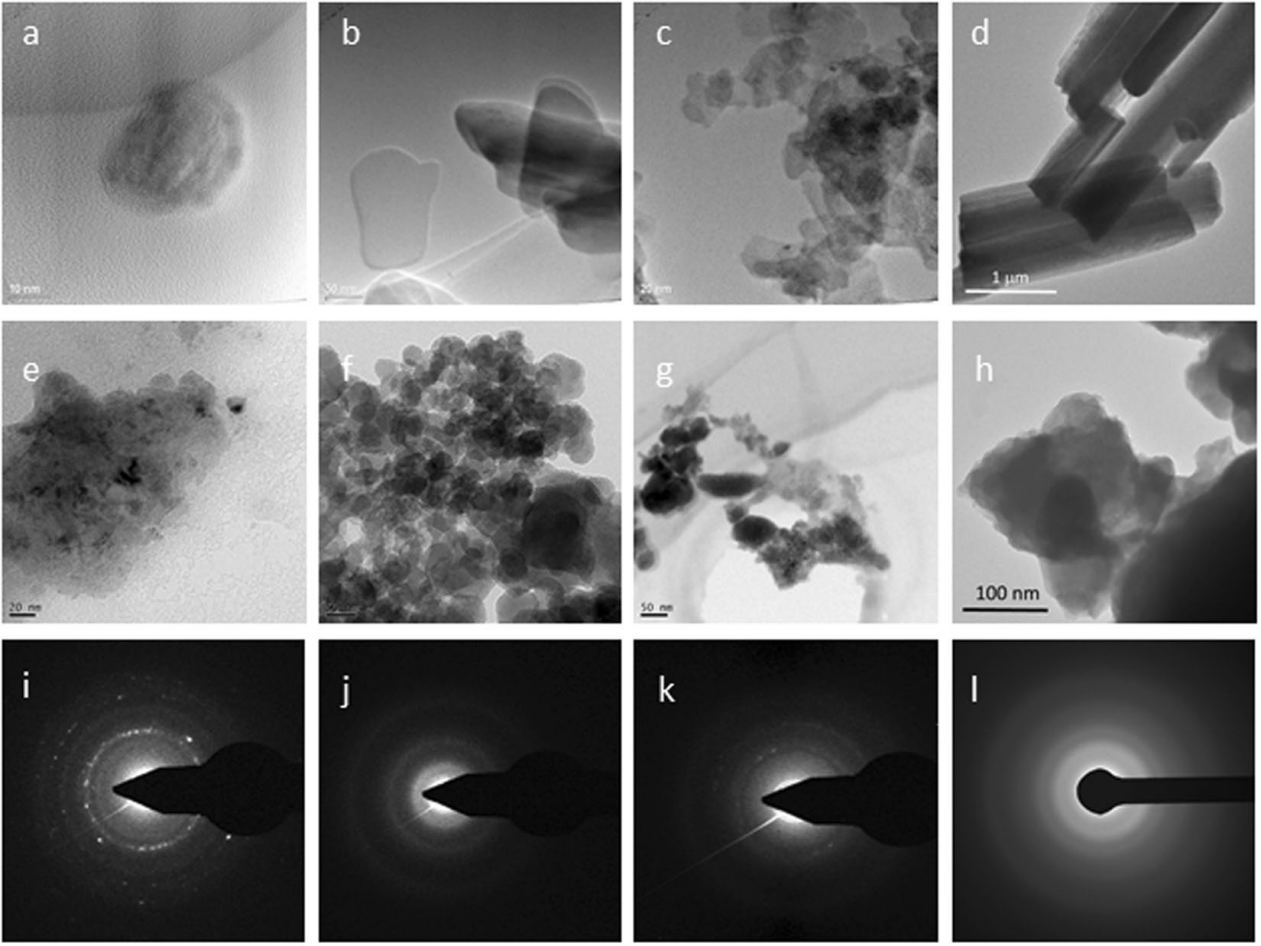
Top row: TEM images of: MCB (**a**) MPB (**b**) MWB (**c**) MVB (**d**). Mid row: TEM images of: MCA (**e**) MPA (**f**) MWA (**g**) MVA (**h**). Down row: ED analysis of: MCA (**i**) MPA (**j**) MWA (**k**) MVA (**l**). ED analysis of all converted materials reveals the amorphous nature of them.

The microstructure and crystallinity of the material were further examined with TEM and electron diffraction (ED) (Fig. 3). All mother salts showed clear borders on the edge of the particles. However, in the case of MCB sample, an amorphous compound has covered the crystalline core of the particle (Fig. 3a). According to the chemical instability of the manganese carbonate phase, this phase has the potential to be converted to oxide. Therefore, this amorphous shell can be attributed to an amorphous oxide phase, which has covered the crystalline manganese carbonate core. An amorphous background in the XRD pattern of this material validates this hypothesis.

HRTEM images of converted materials combined with an ED analysis revealed that upon water electrolysis, all investigated salts converted to assembled amorphous phases, which were appeared as amorphous rings in the ED images.

**Figure 4:**
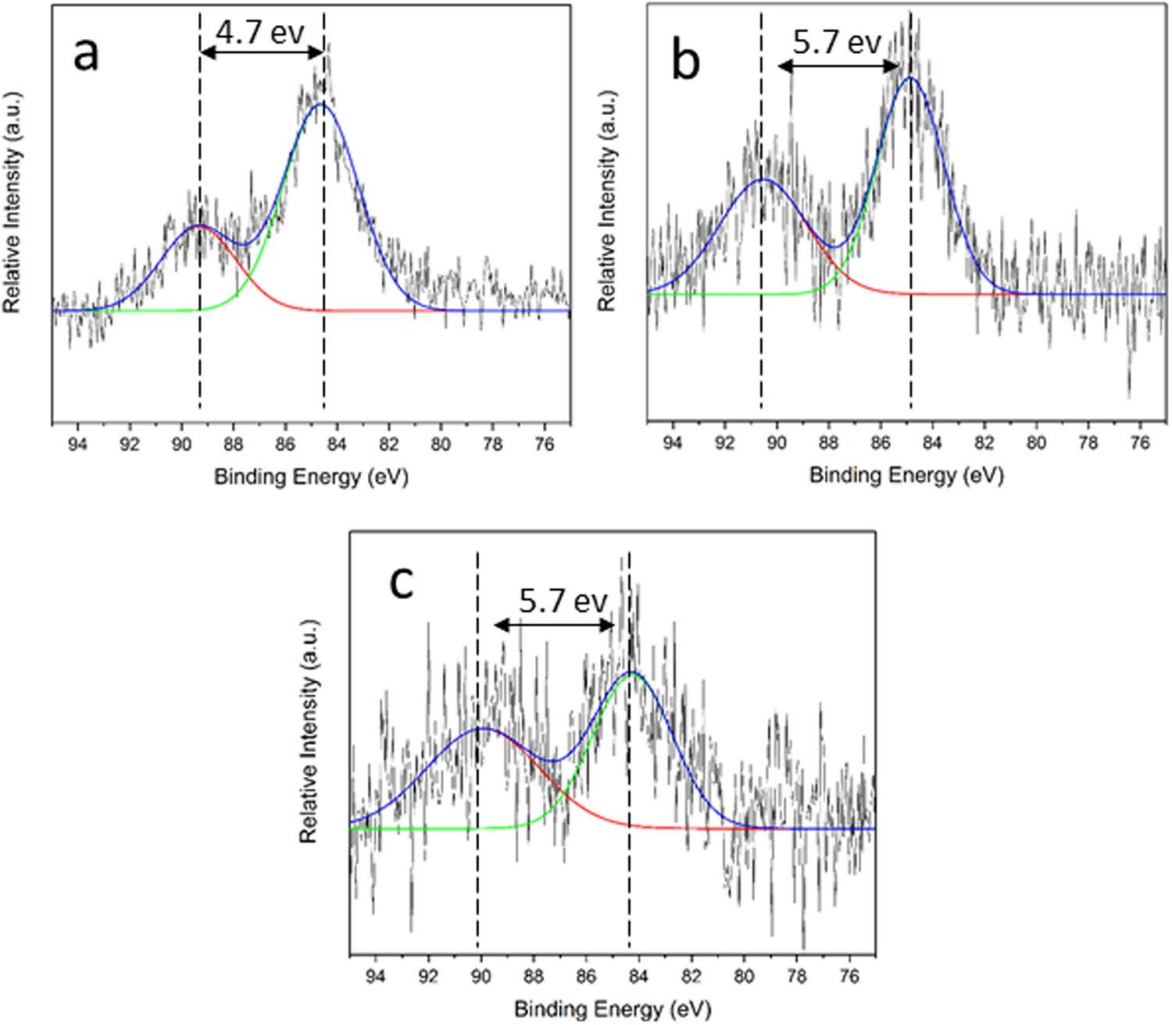
X-ray photoelectron spectra of Mn3s for the converted materials: MCA (**a**) MPA (**b**) MWA (**c**). ΔE 3s for all converted materials is less than 6.0 eV, which indicates oxidation of Mn(II) sites toward higher oxidation states in the converted materials.

In the next step, we used XPS to investigate the probable oxidation of the original salts to manganese oxide (Fig. 4). Therefore, we utilized Mn 3 s peak to distinguish the oxidation state of Mn^41^. Coupling of the non-ionized 3 s electron with 3d valence band electrons in Mn species leads to a splitting which its magnitude can be used to find the oxidation state of it. According to Fig. 4, the ΔE 3 s for all samples after the operation in the water electrolysis is around 4.7, 5.7, 5.7 eV for MCA, MPA, and MWA, respectively. To further investigation of oxidation state and chemical environment of Mn species, we examined the MnWO_4_ sample before and after the conversion with XAS method, as well.

**Figure 5:**
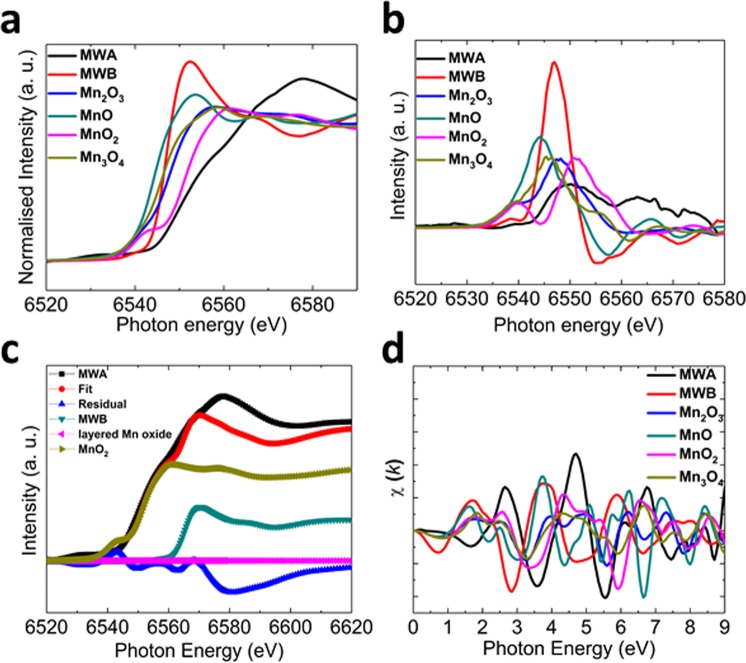
XANES (**a**) and first derivative spectra of MWA and MWB along with references. (**b**) Linear combination fitting of MWA while considering MWB, layered Mn oxide and MnO_2_ as components. (**c**) *k*-weight EXAFS spectra of various materials. (**d**) In comparison to MWB, the white-edge of MWA has shifted to higher energies, which confirms higher oxidation states of manganese sites in this material. A linear combination fitting showed that MWA consists of 72% MnO_2_ and 28% MWB.

XANES and its first derivative show presence of Mn ions in higher oxidation state for MWA compared to MWB (Fig. 5). A linear combination fitting shows that MnO_2_, MWB, and layered Mn oxide exist almost 72, 28 and 0% in MWA

**Figure 6:**
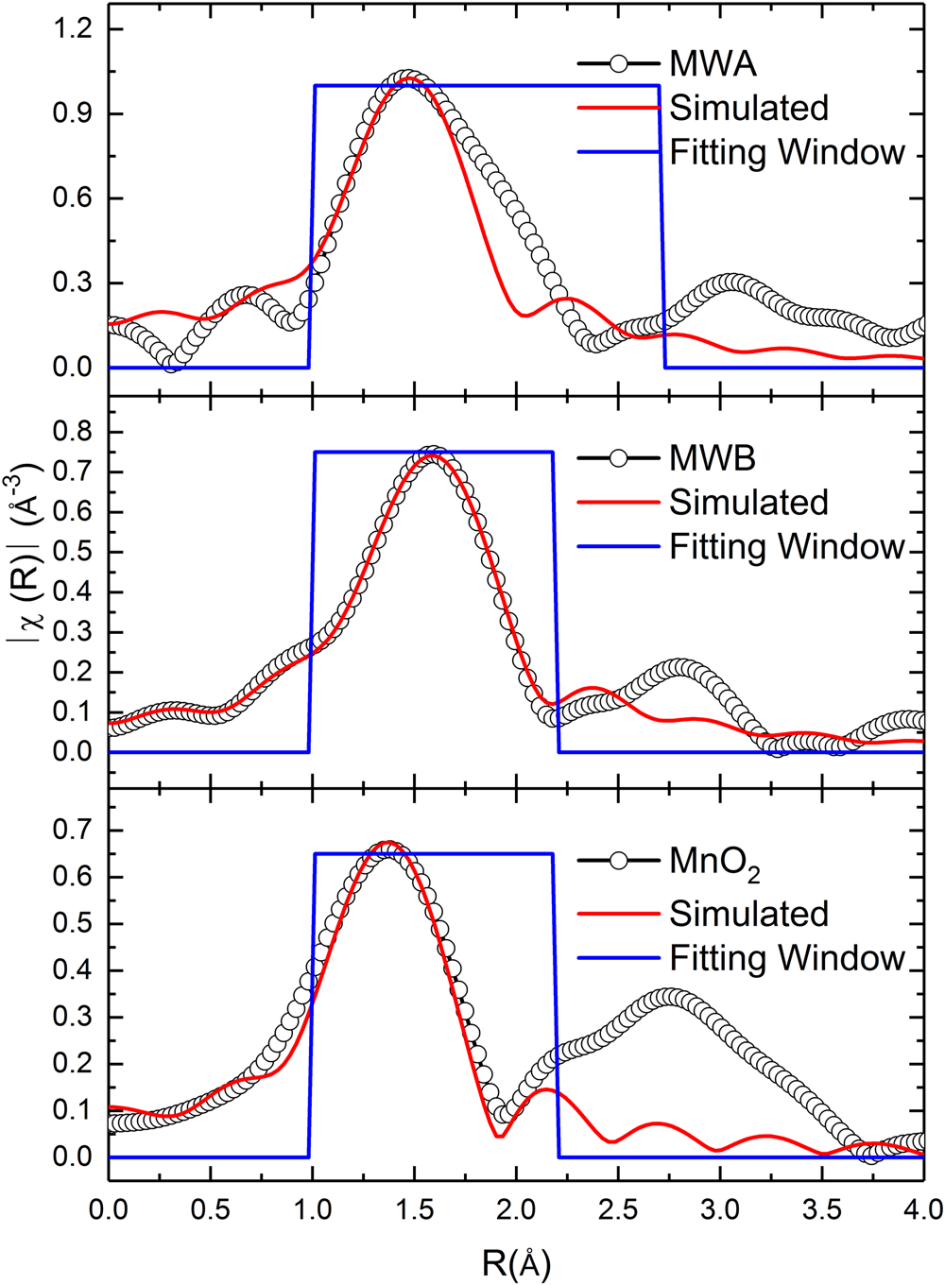
Simulated non-phase corrected Fourier Transform of EXAFS spectra of MWA, MWB and MnO_2_. An inspection of χ(k) spectra envisaged that local coordination of Mn ions in MWB is similar to MnO; however, MWA exhibit similarity with MnO_2_.

An inspection of χ(k) spectra envisaged that local coordination of Mn ions in MWB is similar to MnO; however, MWA exhibit similarity with MnO_2_ (Fig. 6).

Further, simulation envisaged that Mn-O shell is different in MWB and MWA as clear from the Fourier Transform of EXAFS spectra. Co-ordination number and bond lengths have been shown in Table 3.

**Table 3:** Co-ordination number (N) and bond-lengths (R) of Mn-O shell of various materials.

Materials	N	R (Å)
MWA	2.6	1.93871
MWB	3.5	2.11517
MnO_2_	6	1.86266

**Figure 7:**
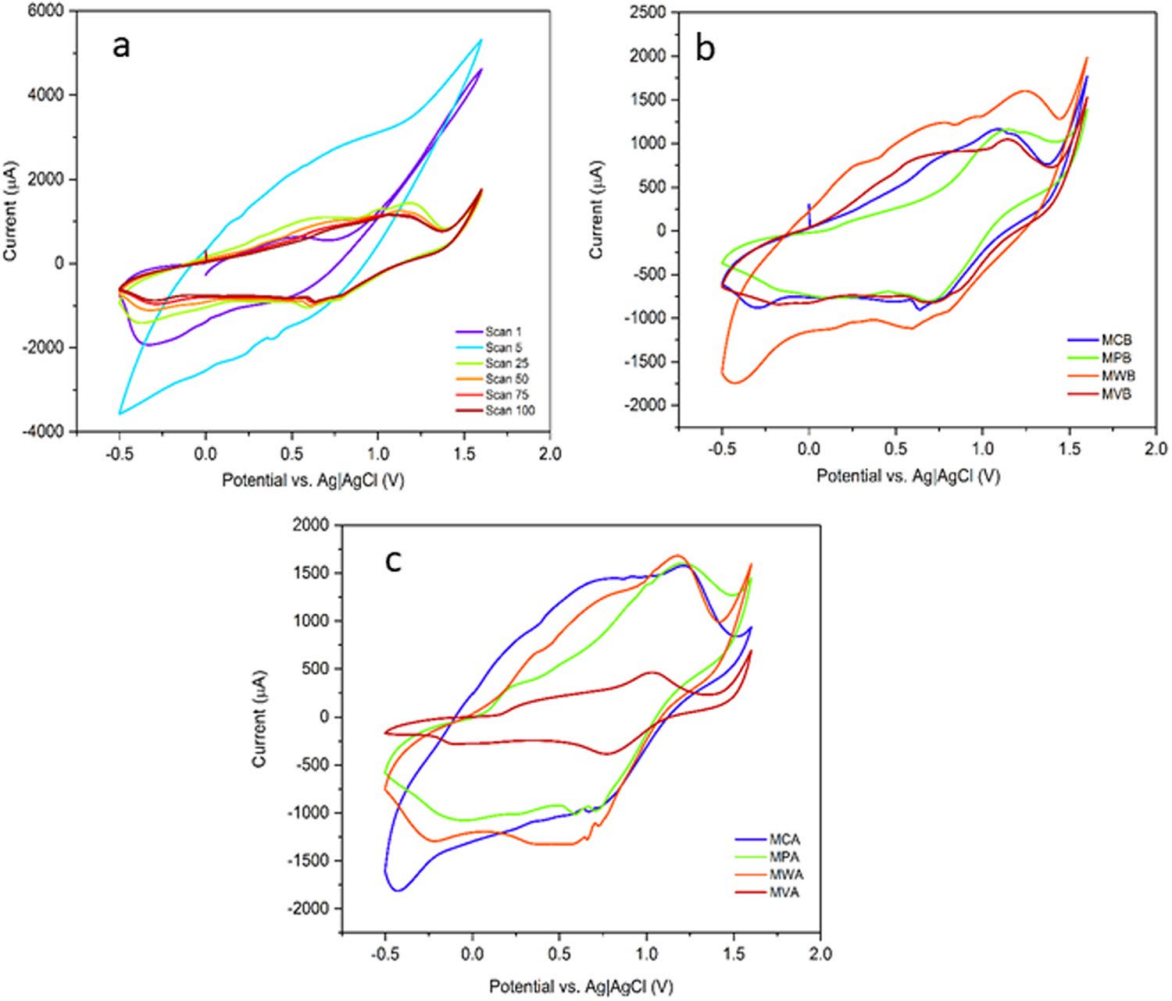
Continuous CV of MCB (**a**) and 100^th^ CV of all original salts. (**b**) CV of the converted materials in the water electrolysis condition. (**c**) A three-electrode system including an FTO slide, Pt foil as a counter electrode and Ag|AgCl|KCl_sat_ as a reference electrode was applied for investigation of the electrochemical properties of the catalyst (room temperature, LiClO_4_ (0.25 M), pH = 6.3, scan rate = 50 mV · s^−1^). Continuous CV of the mother salts confirmed their instability under electrochemical water oxidation conditions. A progressive manganese leakage into solution in the form of [Mn(H_2_O)_6_]^2+^ and its re-oxidation to manganese oxide, under the water oxidation conditions, might provoke a continuous change of surface composition in these materials.

Furthermore, the catalytic stability of these manganese salts was tested through continuous cyclic voltammetry (CV). Consequently, a suspension of the compounds was dispersed on the surface of a bare FTO/glass substrate and was fixed with an ethanolic solution of Nafion. The electrodes were subjected to continuous CV (Fig. 7a). After 5 cycles, an oxidation peak was observed at voltages >1.2 V, for all samples (Figs S6–S9). This peak could be related to the oxidation of manganese ions. CVs of the fresh and the converted compounds are displayed in Fig. 7b,c. Figure 7a, shows that after continuous CV within −0.3–1.9 V, the samples electrochemically behave very similar to amorphous manganese oxides rather than the other phases of manganese oxides, in the same experiment condition^42^. The CV of the original salts in the 100^th^ cycle showed a broad peak within 0.7–0.9 V which was centered at around 0.85 V. This broad peak is attributed to Mn(III) to Mn(IV) oxidation in amorphous manganese oxide^42^. The extreme broadening of signals due to electronic interactions of Mn sites in the amorphous phase manganese oxide led to this broadening^42^. We attributed the difference in the voltammograms to the different rate of conversion of the samples. Moreover, we also observed that cyclic voltammograms of the converted materials in the convective-suspension-collision system were close to amorphous manganese oxides (Fig. 7c).

**Figure 8:**
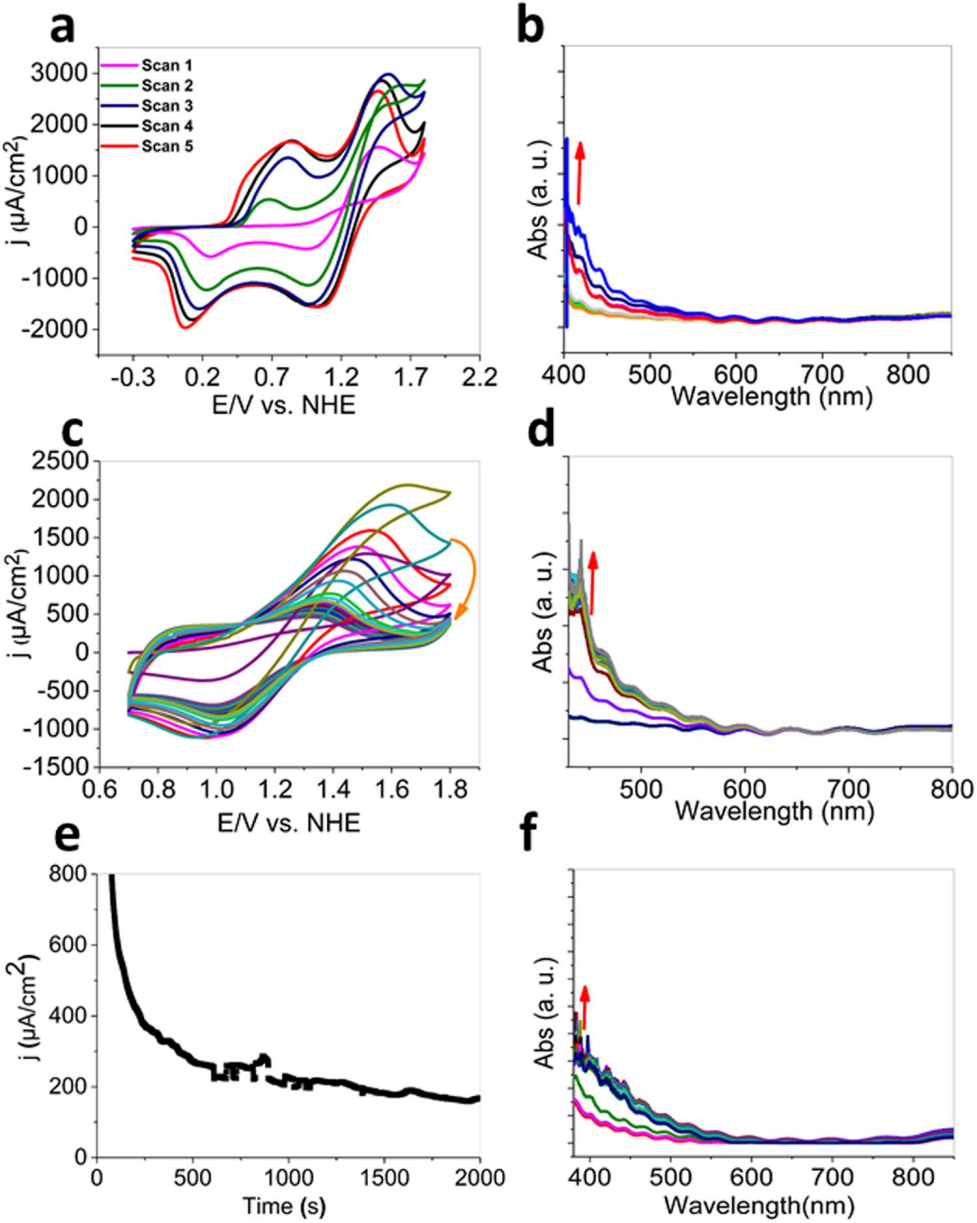
Spectro-electrochemical analysis of MCB under water oxidation using CV (each spectrum after one minute) (**a**–**d**) and amperometry (2.0 vs. NHE). MnCO_3_ was placed on the FTO by Nafion. 1.0 mg of MnCO_3_ was dispersed in 50 μL water. Then, the suspension (30 μL) of this mixture was dripped onto the FTO electrode surface and dried at room temperature. Eventually, 10 μL of 0.5 wt % Nafion solution was deposited onto the center of the modified electrode. A spectro-electrochemical study on a manganese carbonate-coated electrode showed that upon applying a potential to the electrode, the material was not stable and was converted to a manganese oxide phase. With conversion of carbonate, the color of the electrode changed from yellowish-white to dark-brown and a peak at around 400 nm became to view in the visible spectrum.

To have a deep understanding of surface change during the CV, we used *in-situ* spectro-cyclic voltammetry and spectro-amperometry as a probe to characterize the surfaces of the electrodes during cycling (Fig. 8a–f). Upon application of a CV within −0.3–1.8 V, a broad peak at 380–600 nm appeared due to the oxidation of Mn(II) ions in the manganese carbonate phase (Fig. 8a,b). This new peak is similar to those observed previously for manganese oxide films in an aqueous electrolyte^42^. With taking the shape of this peak, the color of the electrode changed from yellowish-white to dark-brown. The peak could be related to the transition between the valence and conduction bands attributed to excitation from an O2p to Mn3d state but with some Mn3d to the Mn3d character^43^. Manganese carbonate with Mn(III) or Mn(IV) sites has not been reported, so the newly formed phase on the surface of the electrode could have a manganese-oxide based structure. We observed similar spectro-electrochemical behavior for the MCB material during continues CV in a 0.7–1.8 V potential window for 20 min (Fig. 8c,d). The water oxidation at 2.0 V indicated such spectrum as well (Fig. 8e,f).

## Discussions

According to Fig. 1, SEM results showed that while the mother salts presented different morphologies, all the converted materials had a very similar view without especial morphology. Therefore, this observation confirmed morphologically instability of the mother salts under water oxidation condition. EDXS results showed that, especially in the case of Mn(VO_3_)_2_ · xH_2_O, the original salts were completely unstable under the harsh condition of water electrolysis, and they were converted to a manganese oxide phase without high-range order. Furthermore, the transformation of the original salts to an amorphous phase was confirmed with XRD method. As it is clear in Fig. 2, well-defined crystalline structures are observed for all original salts. However, unrecognizable diffraction peaks for the converted materials corroborate amorphous phase of these samples. Only in the case of MCA, some broad peaks related to a layered manganese oxide phase are observable in its XRD pattern. More crystallinity of this sample might be linked to the high tendency of the related original salt to degradation. It is hypothesized that in the most beginning of the water- electrolysis reaction the manganese carbonate was converted to completely amorphous manganese oxide and during the reaction the crystallinity of the oxide structure raised.

Additionally, conversion of these manganese salts to an amorphous phase was confirmed with HRTEM combined with ED. Sharp borders of particles in the HRTEM images of all original salts suggested their ordered structure, however, in the case of converted materials, the amorphous nature of the materials was clear using HRTEM and ED studies. Some bright spots were visible in the ED image of the MCA sample, which suggests a partially ordered crystal structure for this sample. However, according to dispersed rings, most of the sample is amorphous. The results by TEM is consistent with the XRD results of the MCA sample in Fig. 1, which a few broad peaks were clear on an amorphous background.

To get more details on the possible oxidation of the investigated salts after water -oxidation reaction, we used XPS method. For all samples, the value of ΔE3s is lower than ΔE3s for MnO (the ΔE for Mn(II) in MnO is >6.0 eV)^44^ which indicates oxidation of Mn(II) sites toward higher oxidation states in the converted materials. We further investigated the MWB and MWA samples with XAS method. XANES revealed the main edge of MWA close to MnO_2_ (Fig. 5a) which is further confirmed by the first derivative of XANES spectra (Fig. 5b). Linear combination fitting of the XANES spectra of MWA revealed the contribution of MnO_2_ in the sample (Fig. 5c). *k*-weight EXAFS spectra envisaged that co-ordination of manganese in these two materials is different from each other (Fig. 5d). Mn-O distance is also smaller for MWA (1.94 Å) compared to MWB (2.11 Å). Mn-O distance in MnO_2_ is around 1.89 Å, which is close to MWA sample. These results show that under water-oxidation conditions the sample was not stable and was converted to manganese oxide species.

According to Fig. 7, our results suggest that the mother salts are not stable during continuous CV studies, as well. A progressive manganese leakage into solution in the form of [Mn(H_2_O)_6_]^2+^ and its re-oxidation to manganese oxide, under the water oxidation conditions, might provoke a continuous change of surface composition in these materials^33^. Such effect has also been observed in structure transformation of La_0.65_Sr_0.35_MnO_3_ or crystalline manganese oxides into amorphous manganese oxide, as well^33^. These results suggested that the manganese salts were not stable as the system evolved in time. The applied potential window (−0.3–1.8) was chosen by purpose. The conversion of the original salts in this potential slot showed that decomposition of the initial materials to manganese oxide occurred even in the potentials lower than 2.0 V (the applied potential in the convective-suspension-collision system). On the other hand, irreversible conversion of the original salts to manganese oxide even after sweeping the potential to mines potentials confirmed that the newly formed manganese oxide is a stable phase.

**Figure 9:**
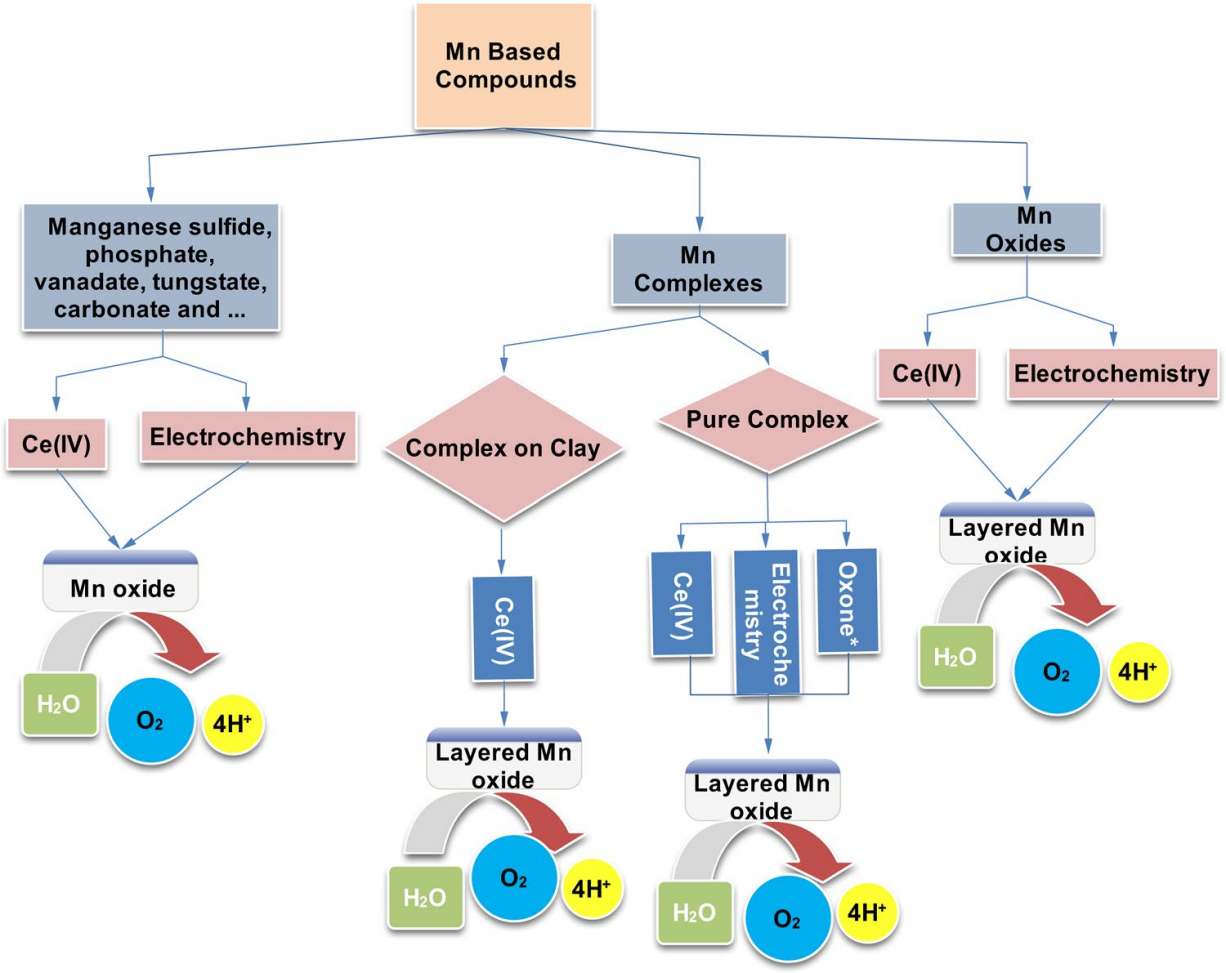
Conversion of different manganese-based materials to amorphous manganese oxide under the chemical or electrochemical conditions. *In the case of Oxone, the molecular-based mechanism was also reported^13^. In the presence of oxidant or under electrochemical conditions, different Mn-based compounds such as Mn-oxides, Mn-complexes and non-oxide Mn-salts are converted to amorphous manganese oxides.

In the light of all the presented data, we suggest that the investigated salts are not stable during the electrochemical water oxidation reaction and they oxidize to a manganese oxide phase. The instability of many manganese salts, manganese complexes, and manganese oxides in the water-electrolysis conditions established that stability of a manganese-based water-oxidizing catalyst is as important as its efficiency and should be carefully considered in the design and synthesize a manganese-based water-oxidizing catalyst (Fig. 9). Such extensive instability for manganese-based Mn compounds is related to the leaking/re-oxidation Mn(II), the decomposition of ligands for Mn complexes, and high stability of amorphous Mn oxide under water oxidation. It seems that, at least, at the end of water oxidation, amorphous manganese oxide is responsible for the catalytic activity of the applied precursors.

## Conclusions

In summary, we studied the conversion of a series of non-oxide Mn-based salts in the electrochemical water oxidation condition, and we observed that all investigated salts were converted to an amorphous manganese oxide phase. This conversion was identified with different methods such as XANES, EXAFS, SEM, EDXS, XPS, HRTEM, spectroelectrochemistry, and electrochemistry. The XRD and SEM results confirmed that although the starting mother salts were crystalline materials with well-defined morphologies, in electrochemical water oxidation condition, they were converted to a manganese oxide phase with very low crystallinity and without any especial morphology. The amorphous phase of the samples was established with ED coupled HRTEM, as well. Furthermore, a CV or amperometry experiment coupled with visible spectroscopy displayed that the salts were instable through the electrochemical water oxidation condition, and a new manganese oxide phase formed during catalytic operation. The visible spectra of the newly formed phase showed a broad peak at 380–600 nm. This peak was related to the transition between the valence and conduction bands attributed to excitation from an O2p to Mn3d state with some Mn3d to the Mn3d character. The oxidation and conversion of the mother salts to a manganese oxide phase was confirmed by XPS and XAS methods, as well. Based on our results and some recent literature reports, we could observe, different Mn-based materials were converted to an amorphous manganese oxide phase. The instability of most Mn-based materials in the water splitting conditions and their conversion to an amorphous manganese oxide phase is a critical point, which should be considered in the design and synthesis of water- oxidizing catalysts and their future applications in scale-up hydrogen production devices.

## Experimental

### Materials and methods

The manganese salts were prepared by a simple method. Firstly, an aqueous solution of MnCl_2_.4H_2_O was added to a prepared solution containing the stoichiometric amount of Na_2_WO_4_ or Na_2_CO_3_ or NH_4_VO_3_ or Na_2_HPO_4_ precursors, and the reaction mixture was stirred for 2 hours at room temperature. After completing the precipitation, the compound was filtered. Then the sample was washed repeatedly with distilled water for removal of free ions. The product was dried at 60 °C for 10 h in air atmosphere. Previous reports on MnCO_3_ show that this material is not stable at elevated temperatures^45^. Therefore, to avoid the manganese oxide impurities in the mother salt, this sample was dried at room temperature.

According to the low solubility of NH_4_VO_3_ in water, when this material was utilized as the precursor, the solution was stirred at 70 °C until a clear yellow solution was obtained. Then the aqueous solution of MnCl_2_.4H_2_O was added to it. Afterward, the remaining steps were repeated as mentioned before. In the next step, the stability of the final samples was studied in a convective-suspension-collision system^39^, which is described with detail in the following section.

### Convective-suspension-collision system

A three-electrode system containing two platinum plates was used to study the stability of the compounds in the water electrolysis condition at near neutral pH. Electrodes were dipped into LiClO_4_ (0.1 M) and KCl (1.0 M) solutions as anodic and cathodic electrolytes, respectively. As a convective-suspension-collision technique, 250 mg of the manganese salts were suspended in 10 mL electrolyte (LiClO_4_) under continuous stirring. A constant potential of 2.0 V across the electrodes was applied for 24 h. After 24 h a dark brown solid, which was precipitated on the surface of the anode, was collected. Then, the compound was washed several times with distilled water and was dried at room temperature. In the case of manganese phosphate salt, the reaction was continued for 5 days. The remaining steps were repeated as same as the mentioned details.

### Electrochemical experiments

Electrochemical experiments were performed using an EmStat^3+^ from PalmSens Company (Netherlands). To fabricate the manganese salts modified electrodes, 2.0 mg of the manganese salts or converted materials, which were emerged from the convective-suspension-collision system, were dispreaded in 100 μL of distilled water. Then 40 μL of the resulting suspension was cast onto the surface of an FTO/glass substrate and was allowed to dry with evaporation. In the next step, 10 μL of an ethanolic solution of Nafion was dropped on the surface of the mentioned electrode to attach the compounds to the electrode. For electrochemical characterization, a three-electrode setup was composed of the fabricated electrode, an Ag|AgCl|KCl_sat_ electrode, and a platinum rod, which served as working, reference, and auxiliary electrodes, respectively.

### Characterization

SEM and TEM images were taken using SEM - Zeiss Supra 50 VP and FEI Tecnai G^2^ F20 transmission electron microscope (TF20 200 kV), respectively. The XRD patterns were recorded with an STOE STADI diffractometer (Mo-K_α_ radiation). The high-resolution visible spectra were recorded by a mini spectrophotometer (Pooyesh Tadbir Karaneh (Phystec), Iran). XPS measurements were done by an X-Ray BesTec XPS system (Germany) with an Al K_α_ X-ray source (hν = 1,486.6 eV). EXAFS measurements for the MWB and MWA along with references were performed at 1D XAS KIST (Korea Institute of Science and Technology) beamline, Pohang Accelerator Laboratory, Pohang, South Korea. EXAFS spectra were processed using ATHENA, these spectra were simulated by ARTEMIS using quick first shell fit.

## Supplementary information

SUPPLEMENTARY INFO
