# Supplementary material for: Electrochemical water oxidation by simple manganese salts
Source: Sci Rep. 2019 May 23;9:7749. doi: 10.1038/s41598-019-44001-z (PMC6533286; doi:10.1038/s41598-019-44001-z)
Supplement: Supplementary file 1 — SUPPLEMENTARY INFO [file 41598_2019_44001_MOESM1_ESM.docx]

**Electrochemical water oxidation by simple manganese salts**

Sima Heidari,^a^ Jitendra Pal Singh,^b^ Hadi Feizi,^c^ Robabeh Bagheri,^d^ Keun Hwa Chae,^b^ Zhenlun Song,^d^ Maasoumeh Khatamian^a^ and Mohammad Mahdi Najafpour ^c,e,f*^

*^a^Department of Inorganic Chemistry, Faculty of Chemistry, University of Tabriz, Tabriz, Iran*

*^b^Advanced Analysis Center, Korea Institute of Science and Technology, Seoul 02792, Republic of Korea*

*^c^Department of Chemistry, Institute for Advanced Studies in Basic Sciences (IASBS), Zanjan, Iran*

*^d^Surface Protection Research Group, Surface Department, Ningbo Institute of Materials Technology and Engineering, Chinese Academy of Sciences, 519 Zhuangshi Road, Ningbo 315201, China*

*^e^Center of Climate Change and Global Warming, Institute for Advanced Studies in Basic Sciences (IASBS), Zanjan, Iran*

*^f^Research Center for Basic Sciences & Modern Technologies (RBST), Institute for Advanced Studies in Basic Sciences (IASBS), Zanjan 45137-66731, Iran*

*Corresponding Author:* *E-mail: mmnajafpour@iasbs.ac.ir*

**Figure S1.** XRD patterns of MCB.

**Figure S2.** XRD patterns of MPB.

**Figure S3.** XRD patterns of MVB.

**Figure S4.** XRD patterns of MWB.

**Figure S5.** XRD patterns of MCA.


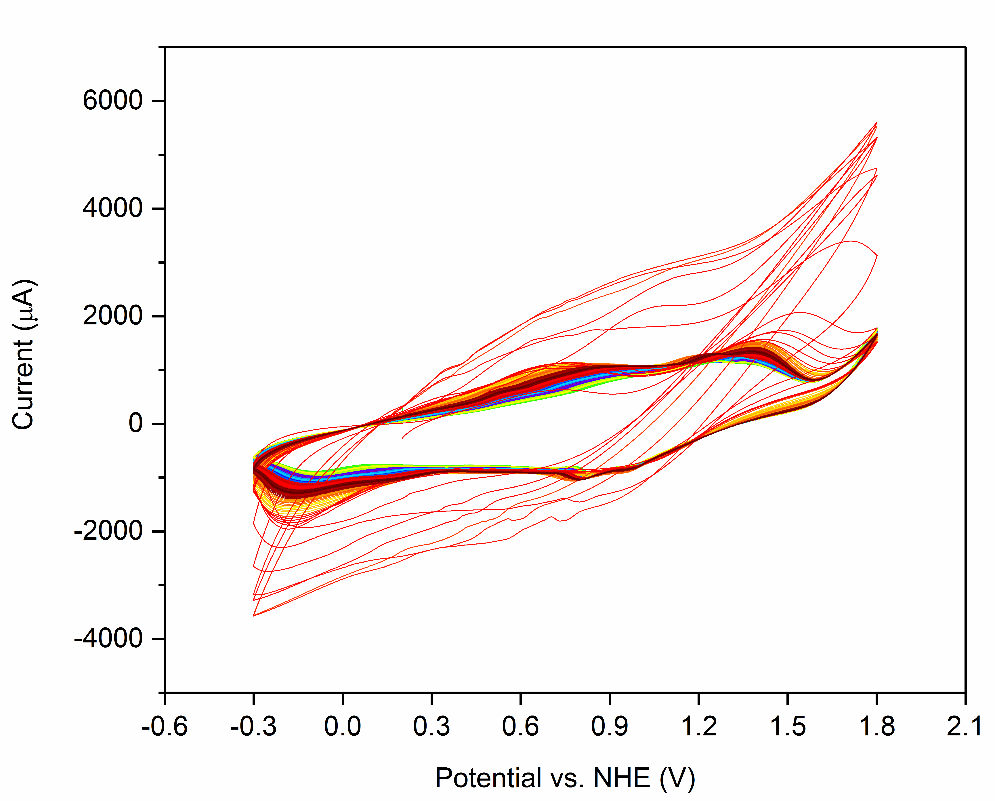


**Figure S6.** Continuous CV of MCB.


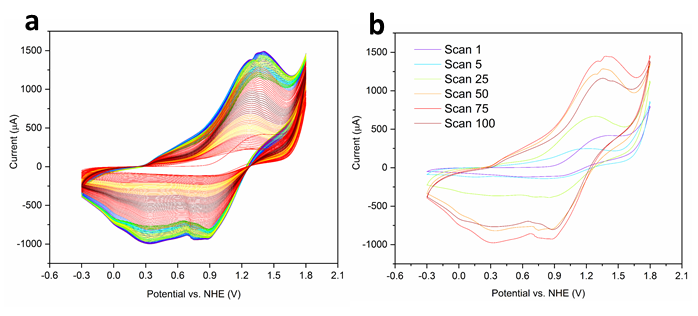


**Figure S7.** Continuous CV of MPB (a) 5 selected cycles (b).


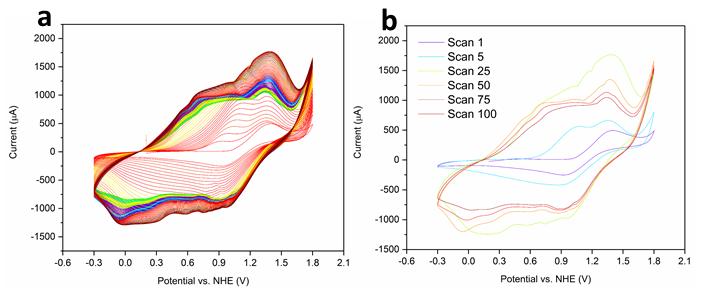


**Figure S8.** Continuous CV of MVB (a) 5 selected cycles (b).


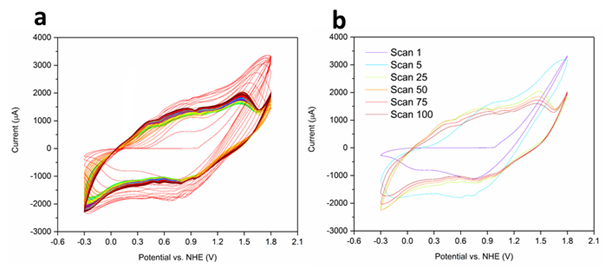


**Figure S9.** Continuous CV of MWB (a) 5 selected cycles (b).


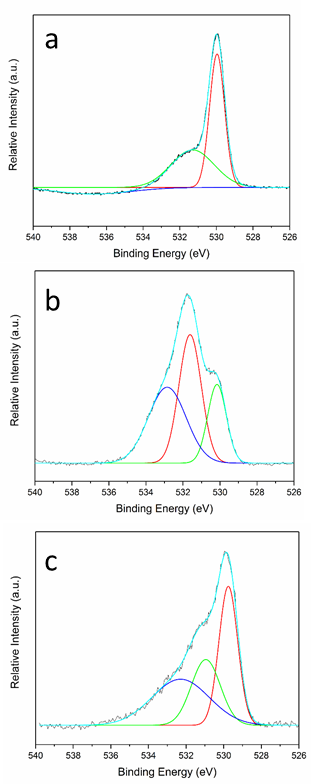


**Figure S10.** X-ray photoelectron O1s spectra of MCA (a), MPA (b), MWA (c).
